# Circuit training intervention for cognitive function, gut microbiota, and aging control: study protocol for a longitudinal, open-label randomized controlled trial

**DOI:** 10.1186/s13063-025-08807-9

**Published:** 2025-03-18

**Authors:** Keishi Soga, Michio Takahashi, Akari Uno, Takamitsu Sinada, Kentaro Oba, Keisei Kawashima, Yasuko Tatewaki, Taizen Nakase, Yasuyuki Taki

**Affiliations:** 1 Smart Aging Research Center, Tohoku University, 4-1 Seiryo-Machi, Aoba-Ku, Sendai, 980-8575 Japan; 2 Department of Aging Research and Geriatric Medicine, Institute of Development, Aging and Cancer, Tohoku University, Sendai, Japan; 3 Department of Medical Sciences, Graduate School of Medicine, Tohoku University, Sendai, Japan

## Abstract

**Background:**

Long-term exercise is increasingly considered an effective strategy to counteract cognitive decline associated with aging. Previous studies have indicated that circuit training exercises integrating aerobic and resistance modalities positively affect cognitive function. Furthermore, a growing body of evidence suggests that long-term exercise alters the gut microbiota, leading to an optimal environment for cognitive enhancement. Recent empirical evidence suggests that exercise plays a significant role in modulating aging-control factors at the protein level. Although the interaction between exercise and cognitive function is multifaceted, most studies have only examined a direct pathway from exercise to cognitive function. Therefore, this study aims to elucidate the effects of long-term circuit training on cognitive function through a comprehensive analysis of factors such as gut microbiota and proteins related to aging control.

**Methods:**

A total of fifty-one participants will be randomly assigned to either the circuit training or waitlist control group. The intervention group will participate in a circuit training program developed by Curves Japan Co., Ltd. two to three times weekly for 16 weeks. The control group will continue their usual daily routines without participating in any new active lifestyle program. The participants will undergo cognitive assessments at baseline and after the intervention. Fecal and blood samples for protein analysis will be collected before and after the intervention. The effect of exercise on cognition will be analyzed by comparing the measured outcomes before and after the intervention. The associations among these outcomes will be assessed using a linear mixed model and structural equation modeling approaches.

**Discussion:**

This study aims to provide the first insights into the comprehensive effects of exercise on cognitive function from the perspectives of gut microbiota and aging control. The findings are expected to contribute to improving brain health and combating age-related cognitive decline. Furthermore, the findings may help establish new guidelines for future studies on the relationship between exercise and cognitive function.

**Supplementary Information:**

The online version contains supplementary material available at 10.1186/s13063-025-08807-9.

## Administrative information

**Table:** 

Title {1}	Circuit training intervention for cognitive function, gut microbiota, and aging control: Study protocol for a longitudinal, open-label randomized controlled trial
Trial registration {2a and 2b}	University Hospital Medical Information Network Clinical Trials Registry (UMIN-CTR), (UMIN-CTR: UMIN000053937) Registered: 21 March 2024 https://center6.umin.ac.jp/cgi-open-bin/ctr/ctr_view.cgi?recptno=R000061564 Additional WHO Trial Registration Data Set items not described in the protocol are presented in Supplemental Table 1
Protocol version {3}	Version 2.0, June 2024
Funding {4}	This study is supported by the fund of Curves Japan Co., Ltd
Author details {5a}	Keishi Soga, Michio Takahashi, Akari Uno, Takamitsu Sinada, Kentaro Oba, Keisei Kawashima, Yasuko Tatewaki, Taizen Nakase, Yasuyuki Taki
Name and contact information for the trial sponsor {5b}	Yasuyuki Taki 4–1 Seiryo-Machi, Aoba-ku, Sendai, 980–8575, Japan
Role of the sponsor {5c}	Checking study progress and implementing exercise interventions

### Introduction

#### Background and rationale {6a}

Age-related cognitive decline is a concern among older adults. As approximately 20% of older adults over the age of 65 have mild cognitive impairments in developed countries [1], prevention of cognitive decline is an urgent issue. Long-term exercise has been suggested as a means of supporting healthy cognitive function as people age. Physical exercise changes brain volume and enhances cognitive performance [2]. Exercise-related growth factors (i.e., the brain-derived neurotrophic factor [BDNF] and insulin-like growth factor [IGF-1]) are thought to contribute to neurogenesis and neural plasticity, leading to increases in specific brain volumes and improvements in cognitive performance [3, 4].

The connection between long-term exercise and cognitive function appears to involve the gut microbiota [5]. This relationship is thought to be due to the regulatory effect of exercise on gut bacteria, which positively influences the growth factors essential for cognitive health. Understanding these mechanisms is crucial to clarify the preventive effects of exercise on age-related cognitive decline. In terms of age-related changes, previous studies have indicated that exercise enhances the activation of aging control factors, such as the mechanistic target of rapamycin (mTOR) in animal models [6]. This increase in activation leads to the recovery of damaged cells and neural plasticity [7]. Considering the crosstalk between gut microbiota and mTOR-related pathways [8], investigating this relationship within the framework of exercise offers valuable insights into how physical activity can prevent cognitive decline associated with aging.

Existing research on the effects of long-term exercise on cognitive function has divided the exercise modality into two main types (i.e., aerobic exercise and resistance training), reaching a consensus that long-term physical exercise improves executive and memory functions [9–12]. Executive functions, described as higher-order cognitive functions for achieving internal aims by regulating behavior and thoughts, include inhibitory control, working memory, and cognitive flexibility [13]. Previous studies have suggested that long-term aerobic or resistance exercise induces enhanced functions of BDNF and IGF-1 [14, 15].

However, it remains uncertain whether the mechanisms of circuit training exercises align with previous findings on aerobic or resistance exercise. Circuit training integrates the advantages of both aerobic and resistance exercises and offers a multifaceted approach to physical fitness. Aerobic exercises enhance cardiovascular fitness, while resistance exercises increase muscle strength and size. Cardiovascular fitness, muscle strength, and muscle size are linked to improved cognitive function [16–19]. Considering that circuit training encompasses both aerobic and resistance exercises, it may effectively harness the positive effects of these exercises on cognitive function. Therefore, exploring the effects of circuit training on cognitive functions is a valuable pursuit. Regarding the effects of circuit training exercises, including both aerobic and resistance exercises, on executive and memory function, a 4-week program of exercise training is suggested to improve the cognitive performance of executive and memory functions [20]. However, no study has yet examined the effects of long-term circuit training on executive and memory functions from a comprehensive perspective of gut bacteria and the control of aging.

Most studies investigating the effects of long-term exercise on the gut microbiota have employed an aerobic exercise modality [21–25]. Exercise has diverse effects on the gut. The beneficial effects are suggested to elicit the secretion of short-chain fatty acids (SCFAs), which play a role in communicating with the brain through immune and vagal pathways called the microbiota-gut-brain axis [5, 26, 27]. This interaction stimulates growth factors and neurotransmitters through sympathetic or parasympathetic pathways by increasing SCFA levels and improving the immune system [28]. Furthermore, previous studies have shown that aerobic and resistance exercises alter gut microbiota diversity and composition [29]. Gut diversity is related to brain connectivity networks that mediate executive functions [30]. Considering that the gut microbiota is thought to affect the hippocampus and cingulate cortex [27, 31], such exercise-induced alterations in the gut microbiota can be linked to improved executive and memory function. In addition, exercise-induced dynamic changes in gut composition enhance communication between the gut and brain, leading to improvements in executive and memory function. Thus, the interaction between exercise and the gut might protect against age-related deterioration of executive and memory functions.

A multi-omics analysis can enrich our understanding of the hidden effects of physical exercise on the brain within the field of neuroscience. This method, which involves the analysis of proteins from small blood samples, facilitates the detection of multifaceted factors, such as inflammatory cytokines and growth factors that promote neurogenesis [32]. Consequently, this approach is considered pivotal for enabling researchers to examine the intricate interactions among various biological pathways and systems. This enhanced capability significantly broadens and deepens the knowledge base in the fields of exercise science and neuroscience, providing a more comprehensive understanding of the biological effects of physical activity on the brain [33]. A notable aspect of omics analysis is its ability to identify the factors that influence the aging process. Recent studies revealed age-related signal transactions, suggesting that longevity is partially dependent on mTOR and SIRT activation [7, 34]. Through increased mTOR signaling via BDNF secretion, damaged neural cells are directed to recuperate [7]. Physical exercise might improve neural plasticity and cognitive performance by stimulating mTOR signaling pathways, resulting in enhanced mitochondrial function, which is thought to be essential for promoting neurogenesis through brain metabolism [35, 36]. Exercise-induced muscle activity increases mitochondrial density and SIRT activity [37]. A recent meta-analysis examining the effects of exercise on SIRT has indicated that relatively high-intensity and resistance exercises might be effective in increasing SIRT levels [38].

Furthermore, as an exploratory investigation, proteomic analysis, one of the omics analyses, enables the measurement of more than thousand proteins in blood samples. Recent studies have used omics analysis to identify the risk factors for cognitive dysfunction and psychiatric diseases [39]. Although a wealth of studies has indicated an underlying mechanism by which long-term exercise induces improvements in cognitive function, such as BDNF and IGF-1, it is possible that yet-unrevealed factors could contribute to the exercise-induced enhancement of cognitive function. The results of omics analysis are influenced by sex differences [40, 41]. This methodological study indicates that research combining data from both sexes is inadequate for analyzing datasets that include sex as a covariate. As this study employs an intervention approach and does not intend to analyze large sample sizes, only women will be recruited to explore the association between circuit training and cognitive function using omics resources. Understanding these mechanisms will provide new insights into the research field of exercise and the brain, potentially leading to the discovery of novel mechanisms through which long-term exercise supports brain health.

Circuit training may offer distinctive advantages over single-mode exercise by simultaneously activating multiple physiological adaptation pathways in a time-efficient manner. Unlike traditional studies that typically implement 60-min or longer sessions of either aerobic or resistance training, our 30-min circuit training protocol is designed to be more accessible and feasible for integration into daily life while maintaining exercise benefits. Moreover, we adopt an exploratory approach to investigate potential underlying mechanisms. Our aim is to examine changes in gut microbiota composition following regular circuit training, postulating that shifts in gut microbiota could influence cognitive function through pathways such as reduced neuroinflammation or altered neurotransmitter availability in the gut-brain axis. Additionally, we conduct exploratory analyses of protein expression changes to identify key proteins potentially involved in exercise-induced cognitive enhancement, including those related to neurotrophic factors or metabolic processes.

### Objectives {7}

Although accumulating evidence has demonstrated the positive impact of aerobic and resistance training by clarifying the underlying mechanisms, the positive effects of circuit training on the brain and cognitive performance remain unclear. Thus, this study aims to clarify the effects of long-term circuit training on executive and memory functions from a comprehensive perspective in terms of the gut microbiota, cytokines, and proteins involved in neuroplasticity and aging control. We hypothesize that long-term circuit training improves executive and memory functions. Furthermore, this relationship is likely mediated by the gut microbiota and aging control factors through proteomic analysis. Our central hypothesis is that exercise intervention will modulate gut microbiota composition and metabolite (e.g., increasing short-chain fatty acid [SCFA]-producing bacteria), leading to changes in key aging-related proteins (e.g., SIRT, mTOR) and cytokines (both pro- and anti-inflammatory), which in turn will positively affect cognitive function and neuroplasticity. We hypothesize the following mechanism:

1. Exercise → shifts in gut microbiota diversity/composition → increased SCFA levels → enhanced neuroplasticity and cognitive function;
2. Exercise → altered cytokine profiles → modulation of SIRT/mTOR pathways → enhanced neuroplasticity and cognitive function.

Additionally, we hypothesize that the circuit training group will show superior cognitive outcomes compared with the control group. In addition, we will explore the underlying mechanisms through gut microbiota and proteomic analyses, as well as psychometric assessments of psychological factors.

### Trial design {8}

An open-label randomized controlled trial will be conducted.

## Methods: participants, interventions, and outcomes

### Study setting {9}

This study will be conducted in Tohoku University, Japan.

### Eligibility criteria {10}

The following inclusion criteria will be applied in this study:

- 40- to 74-year-old Japanese native speakers;
- Females only;
- Able to participate in this study two to three times a week for 16 weeks; and.
- Provide informed consent and agree to participate in this study.

Exclusion criteria:

- Subjects unable to provide informed consent;
- Subjects having nervous system diseases (e.g., dementia);
- Subjects taking medication that potentially disturb cognitive function (e.g., benzodiazepines, antidepressants, anticonvulsants);
- Subjects with any of the following serious medical conditions or those currently taking medications that could cause such conditions (to be verified by the co-investigator during the consent explanation):

Those with a history or current diagnosis of renal impairment (including severe chronic kidney disease), liver impairment, heart disease, severe hypertension (systolic blood pressure of 180 mmHg or higher and diastolic blood pressure of 110 mmHg or higher), stroke, psychiatric disorders, epilepsy, autoimmune diseases, multiple sclerosis, systemic lupus erythematosus (SLE), and those currently taking the following medications: antiepileptics, antidepressants, antipsychotics, antiarrhythmics (limited to those who have been instructed by their physician not to exercise).

- Subjects with severe visual or auditory impairments;
- Subjects who regularly engage in exercise at gyms or sports clubs;
- Pregnant or nursing individuals;
- Subjects in other clinical studies (involving exercise interventions or cognitive function assessments);
- Subjects for whom participation in the study is deemed difficult; and
- Subjects affiliated with the department(s) of the principal investigator or co-investigators (including students, graduate students, and staff).

### Who will take informed consent? {26a}

The researchers will explain to participants the purpose of this study, including the inclusion and exclusion criteria, benefits, and risks. The researchers will carefully explain the informed consent process to participants in person along with the relevant documents, highlighting that their participation is voluntary and that they can withdraw from the study at any time by submitting a withdrawal form. Once participants confirm their complete understanding of this study, they will fill out an informed consent form approved by the Ethics Committee of the Tohoku University Graduate School of Medicine.

### Additional consent provisions for collection and use of participant data and biological specimens {26b}

The participants will be informed that the data from this study will be used in future studies. This statement is included in the informed consent forms. The obligation of agreement with the statement is not applied to other studies of their participation.

### Interventions

#### Explanation for the choice of comparators {6b}

The participants will be randomly assigned to either the exercise intervention or control group. The Research Randomizer (https://www.randomizer.org/) is used for randomization. Participants assigned to the intervention group will engage in combination circuit training developed by Curves Japan Co., Ltd. Meanwhile, participants assigned to the control group will have to maintain their ordinary lifestyles without starting new active habits (e.g., joining sports clubs). The control group will be used to compare the effects of adopting a new active lifestyle through circuit training with the control group (i.e., maintaining ordinary daily activities). As the primary aim of the study is to assess the impact of exercise initiation on brain health in previously sedentary adults, this design allows for a direct comparison between those who remain sedentary and those who begin regular exercise.

### Intervention description {11a}

**Fig. 1:**
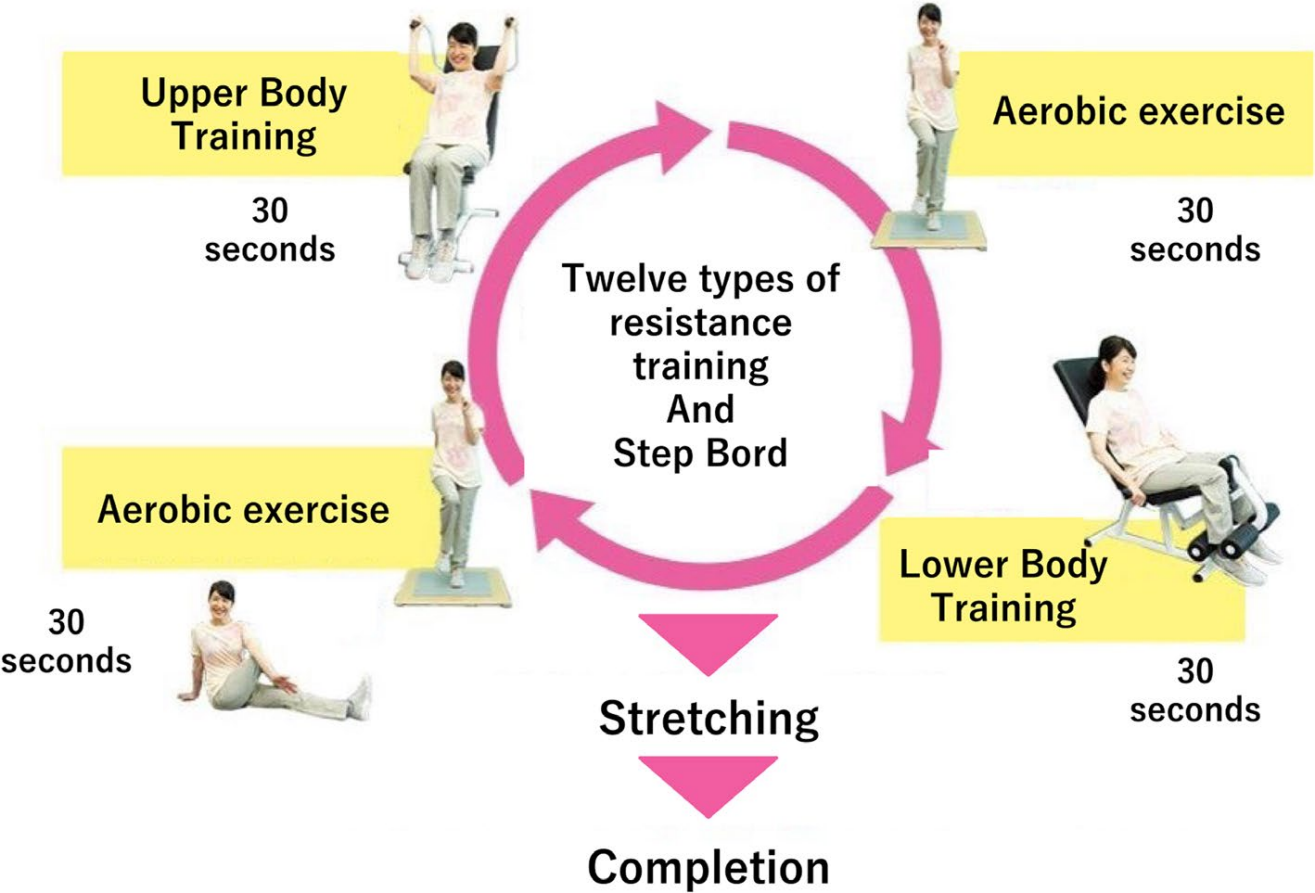
Circuit training procedure

Participants assigned to the intervention group will engage in circuit training 2–3 days per week for 16 weeks. The combined interval training consists of three parts: resistance exercise, aerobic exercise, and stretching. This training modality has been described in previous studies [42]. Each session is approximately 30 min. During the session, participants perform resistance exercises, and during the interval, they perform aerobic exercises, followed by stretching. The participants are required to perform two sets of combined exercises (i.e., resistance exercise + aerobic exercise). The exercise procedure is illustrated in Fig. 1. After the sets of exercises, the participants perform stretching consisting of 12 types of motions (Achilles’ tendon, sole of the foot, thigh, armpit, shoulder, shoulder/upper arm, chest/arm, shoulder/chest/arm, waist, back of knee, base of thigh, and back). A previous study categorized the training intensity as moderate to vigorous level at 60 to 80% of maximal heart rate (HR) during aerobic exercise [43].

Resistance training includes 12 types of exercises (chest press/seated row, squat, shoulder press/lat pull, leg extension/leg curl, abdominal crunch/back extension, lateral lift, elbow flexion/extension, horizontal leg press, pectoral deck, oblique, hip abductor/adductor, and gluteus). Under the instructions of qualified instructors, the participants will perform machine-based resistance training. The machines depend on calibrated pneumatic resistance pistons. In the circuit training, participants will perform as many repetitions as possible in a 30-s time period. During the rest periods, participants will perform floor-based aerobic exercises (e.g., running or skipping in place) on a stepboard.

In the waitlist control group, participants will continue to perform their ordinary daily life activities as usual and not join any new exercise clubs or gyms during the study period. Since one purpose of this study is to evaluate exercise as a supporting tool for brain health in sedentary adults, we will compare the lifestyles of those who remain sedentary with those who begin a new active lifestyle (i.e., long-term combined interval training). Thus, no active control group is recruited for this study.

### Criteria for discontinuing or modifying allocated interventions {11b}

Participants have the right to withdraw from the study at any time after submitting a withdrawal form. The intervention will be discontinued when participants have difficulty maintaining exercise training because of injury or pain.

### Strategies to improve adherence to interventions {11c}

The participants in the intervention group will engage in circuit training sessions alongside regular exercisers. This setup allows for interaction and social bonding, which can enhance the participants’ experience and potentially reduce dropout rates by creating a supportive community environment. Such social connections are crucial, as they provide encouragement and a sense of belonging, which are important factors in maintaining consistent participation in the program. Exercise adherence will be systematically monitored through weekly attendance tracking of circuit training sessions and regular follow-up communication (phone or email) with participants who miss consecutive sessions to verify their well-being and address potential barriers. When applicable, participants will also complete brief workout logs documenting perceived exertion and session details.

### Relevant concomitant care permitted or prohibited during the trial {11d}

Participants in the intervention group will be instructed not to start new long-term physical activities except for the intervention. The participants in the control group will also be instructed not to participate in any new long-term physical activity. No restrictions on medication intake prescribed by a doctor during the intervention period will be conveyed to participants who meet the inclusion and exclusion criteria prior to the intervention. However, the participants will be required to inform the investigators about any medications they are taking so that the potential impact of these medications on cognitive performance can be considered.

### Provisions for post-trial care {30}

The exercise training will be conducted over a 16-week period. Participants will have no restrictions after the completion of this study.

### Outcomes {12}

The investigation presented herein offers a comprehensive examination of the gut microbiota, cytokines, and proteins involved in neuroplasticity and aging control. This study will obtain demographic variables, including year and date of birth, age, sex, educational background, past medical history, present illness history, current medication and supplements, height, and body weight. Executive function will be assessed using the Stroop, flanker, and N-back tasks (1-back and 2-back). Memory function will be assessed using a source-memory task. Questionnaires about psychological characteristics will assess grit (a Japanese translation of the 8-item Short Grit Scale), resilience (the 6-item Brief Resilience Scale-Japanese version), and mindset (the Japanese version of the Self-Theories of Intelligence Scale). Additionally, the Japanese versions of the 6-item Kessler Psychological Distress for Depression and the 10-item Japanese version of the Perceived Stress Scale for measuring perceived stress will be used. This study will assess physical activity using Global Physical Activity, Mood, Constipation, Sleep state, and QOL. The detailed descriptions are provided below. Furthermore, this study will assess physical function by measuring hand grip strength, conducting a 5-m walk test, and evaluating single-leg support.

The primary objective of this study is to elucidate the effects of exercise-induced cytokines and proteins involved in the control of aging on cognitive function. To date, no studies have thoroughly explored these associations. This study aims to provide pioneering insights into the relationship between exercise and brain function, thereby establishing a foundation for future research. As a secondary outcome, an exploratory investigation will examine the relationship between the gut microbiota and protein analysis from blood samples. This approach will enable a deeper understanding of the biological processes involved and may lead to targeted interventions that promote cognitive health.

By combining randomized assignment with multi-level statistical methods, we can:

1. Measure how the exercise intervention alters gut microbiota/metabolite, inflammatory/anti-inflammatory cytokines, and aging-related proteins;
2. Identify the biomarkers or pathways most strongly associated with improvements in cognitive outcomes; and
3. Elucidate possible mediating mechanisms (e.g., exercise → gut microbiota → SCFA → cognition, exercise → SIRT/mTOR → cognition).

The primary outcome of this study is cognitive function, which will be assessed by comparing changes from baseline to 16 weeks after the intervention. We will evaluate four key cognitive domains using standardized tasks. Executive function and attention will be measured using a paper-and-pencil version of the Stroop task, analyzing changes in both reaction time (milliseconds) and error rates. Selective attention and response inhibition will be assessed using a computerized Flanker task, measuring changes in accuracy (percentage of correct responses) and reaction time. Working memory capacity and updating will be evaluated using an n-back task (with *n* = 1 and *n* = 2 conditions), examining changes in accuracy and reaction time. Finally, hippocampal-dependent memory function will be assessed through a source memory task, measuring changes in accuracy of responses. All these measurements will compare performance between baseline (week 0) and the end of the intervention period (week 16). In terms of compliance criteria and statistical analysis of non-compliance, participants must attend at least two sessions per week. Based on previous research [44] demonstrating cognitive improvements with ≥ 70% attendance, we will establish a 70% compliance threshold. For the main analysis, we will include participants who completed both pre- and post-intervention assessments, with missing data handled using the multiple imputation method and a machine learning approach, such as random forests. Furthermore, if notable variation in attendance occurs, we will conduct stratified analyses using the 70% threshold to explore potential dose–response relationships.

### Participant timeline {13}

**Fig. 2:**
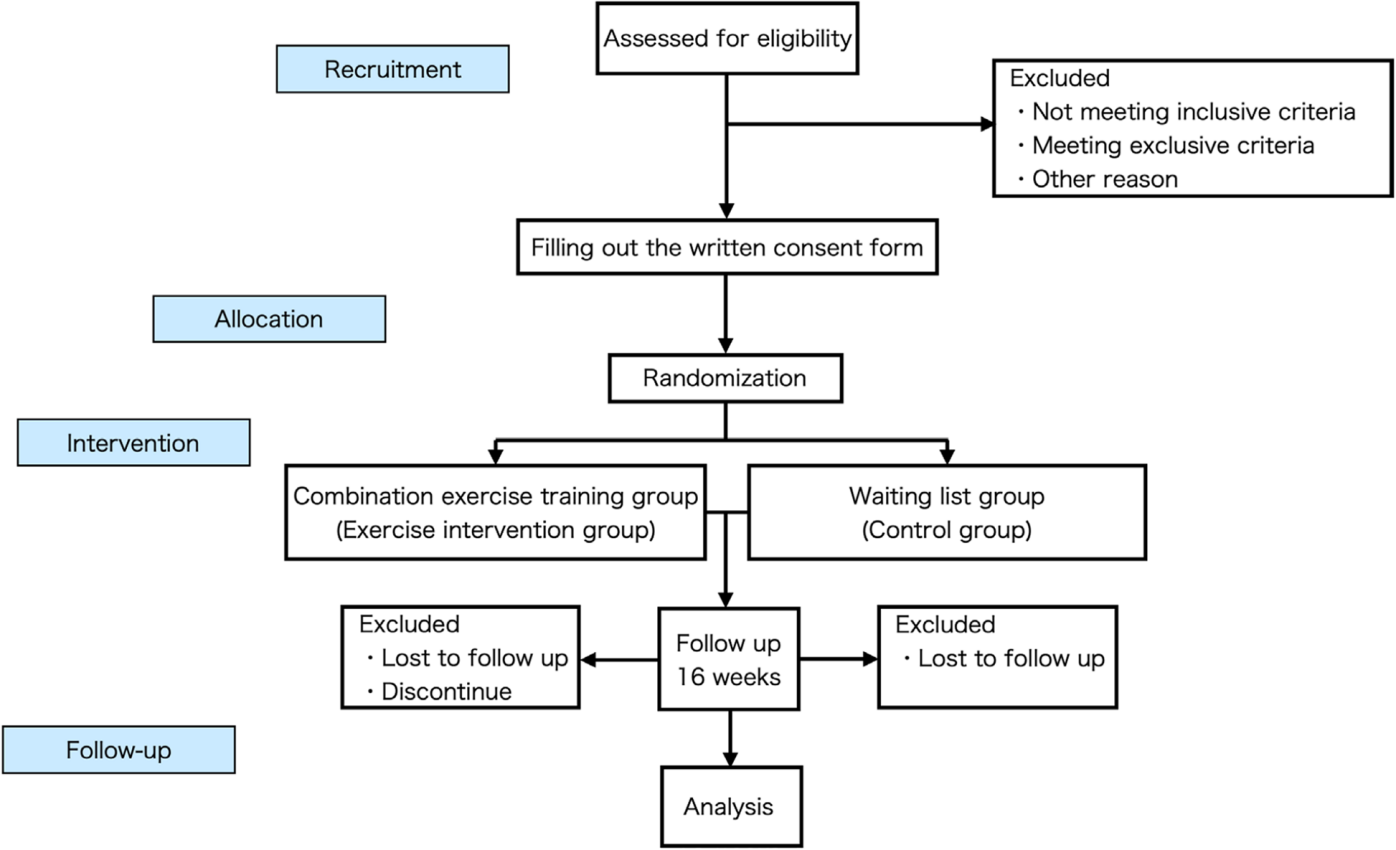
The protocol flow chart and timeline of the study

A flowchart of this study is shown in Fig. 2, and the participant timeline is presented in Table 1. This study will be conducted in accordance with the Standard Protocol Items: Recommendations for Interventional Trials (SPRIT).

**Table 1:**
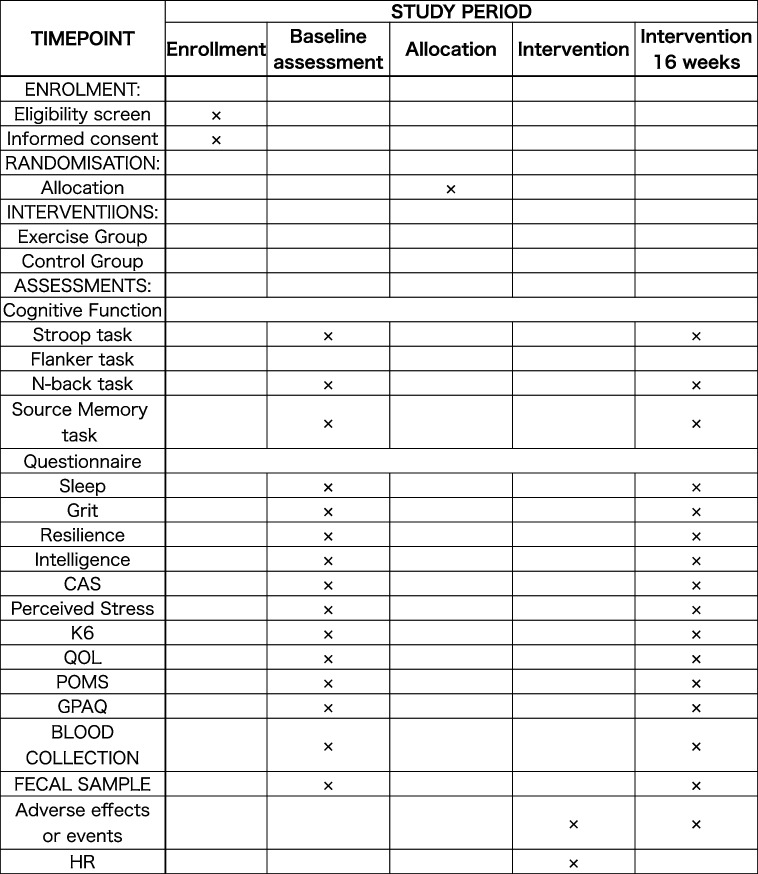
Schedule of enrollment, intervention, and assessment based on SPRIT guidelines

*CAS* Constipation Assessment Scale, *K6* 6-item Kessler Psychological Distress, *QOL* MOS Short-Form 36-Item Health Survey, *POMS*: Profile of Mood States, *GPAQ* global physical activity questionnaire, *HR* heart rate

### Sample size {14}

In this study, 51 participants will be included, with twenty-eight in the intervention group and twenty-eight in the control group. Beyond traditional intervention research, this study conducts a comprehensive protein analysis using omics techniques, enabling the observation of about a thousand proteins. An additional aim of this study is to identify the factors contributing to the exercise-induced enhancement of cognitive function. Previous studies have examined the effects of exercise on the results of omics analysis by recruiting relatively small sample sizes [32]. Considering that omics analysis was conducted in previous studies with a range of sample sizes from 10 to 100 [45], our study is likely capable of performing the experiment even with a relatively small sample size. Based on previous meta-analyses showing effect sizes (d) of 0.25–0.32 for cognitive improvements following multicomponent exercise programs [10], we conservatively estimate a medium effect size (f) of 0.25. Using the analysis of variance framework, the total sample size is calculated with G*Power 3.1 software [46]. The calculation, based on an effect size (f) of 0.25, a power (1 − β) of 0.9, and a significance level (α) of 0.05, indicates a total sample size of 46. Factoring for a potential dropout rate of 10%, the final required sample size will be approximately 51. Furthermore, from the perspective of exercise and gut microbiota, a previous study was conducted with a total sample size of fewer than 40 participants, including both intervention and control groups [22, 23, 47]. Based on the protocol from a previous study, our study can feasibly analyze the effects of long-term circuit training exercises on cognitive function from the perspective of proteome and gut microbiota analyses.

### Recruitment {15}

Participants will be recruited from among the residents of a local area near Tohoku University, as they are required to visit the university for pre- and post-intervention assessments. Furthermore, those assigned to the intervention group will be required to participate in exercise sessions two to three times per week for 16 weeks. Recruitment will be advertised on a local information paper and distributed free of charge to residents. The contact information of this study, including the barcode URL and email address, will be printed in this paper. Interested residents can reach out using the contact details provided, after which we will respond directly to them. Candidates who meet the inclusion criteria will be contacted to arrange a date for obtaining informed consent.

## Assignment of interventions: allocation

### Sequence generation {16a} and Allocation concealment mechanism {16b}

This study will be conducted as an open-label, randomized controlled trial. Thus, the participants will be randomly assigned to the circuit training or wait-list groups in a 1:1 ratio. Simple randomization will be performed using Research Randomizer (www.randomizer.org), a computer-based random number generator. This web-based service will be used to generate the random sequence for participant allocation to either the circuit training or control group. To ensure allocation concealment, the random allocation sequence will be generated only after each participant is deemed eligible and formally enrolled based on the inclusion and exclusion criteria. Participants will be informed of their group assignments to complete the pre-examination.

### Implementation {16c}

The principal investigator will conduct the abovementioned randomization process to ensure that participants meet all inclusion and exclusion criteria. Once a participant is determined to meet the eligibility criteria and is enrolled, the principal investigator will generate the allocation assignment using the computer-based sequence. Participants will be informed of their group assignment only after the enrollment and baseline assessment procedures are completed. This approach ensures that baseline data collection remains unbiased and unaffected by prior knowledge of group assignment.

### Who will be blinded {17a}

Owing to the open-label protocol employed in this study, a blinding process will not applied. After announcing group assignments to the participants upon completion of the pre-examination, both the researchers and participants will be aware of the assigned group.

### Procedure for unblinding if needed {17b}

Not applicable as no blinding will be used in this trial.

### Data collection and management

#### Plans for assessment and collection of outcomes {18a}

In this paper, we list each scale (e.g., Short Grit Scale, Brief Resilience Scale, Self-Theories of Intelligence Scale) along with its psychometric properties, scoring procedures, and references for validity and reliability. Second, we clarify the measurement timeline, indicating that all scales will be administered at baseline and at 16 weeks. Finally, we will ensure consistency between Sect. 18a and the Outcomes section by explicitly mapping each scale to its corresponding study outcome or exploratory variable (e.g., resilience as a psychological characteristic, perceived stress as a mental health indicator).

### Cognitive task

In this study, cognitive function will be assessed using both the pencil-and-paper and computer-based versions. The computer-based version will be conducted using PsychoPy, which uses the Python programming language.

We will use a paper-and-pencil version of the Stroop task, as described in a previous study [48], to assess inhibitory control. This task will include four conditions: two neutral, one Stroop, and one reversed Stroop. The Stroop paradigm comprises four conditions. In Neutral Condition 1, participants see color words (e.g., “RED”) printed in black ink, which serves as a baseline measure of word processing. In the Reverse-Stroop Condition, participants see incongruent color-word combinations (e.g., the word “RED” printed in blue ink) and must select the color meaning of the word (“RED”) rather than its ink color. Neutral Condition 2 presents colored patches (e.g., a red box) where participants mark the corresponding color name, serving as a baseline for color processing. Finally, in the Stroop Condition, participants see incongruent color-word combinations (e.g., the word “RED” printed in blue ink) and must indicate the ink color (“blue”) rather than the word’s meaning. In all conditions, the participants will be instructed to respond as quickly and accurately as possible within 60 s. This task measures the number of correct items.

**Fig. 3:**
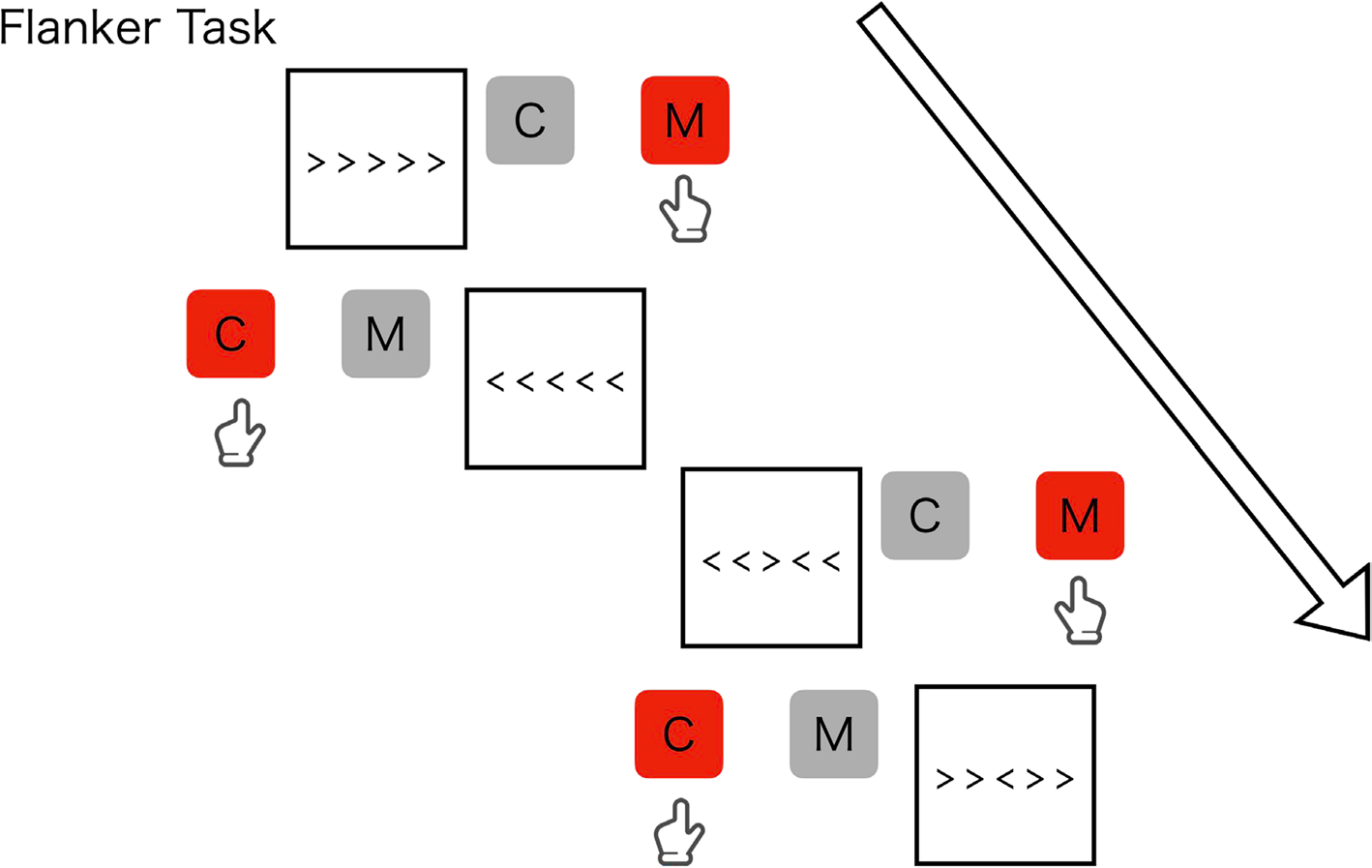
Examples of stimuli and correct judgments in Flanker task

The Flanker task, which incorporates both congruent and incongruent conditions, serves as a method for evaluating inhibitory control through a computer-based implementation (Fig. 3). Participants will be instructed to respond as quickly and accurately as possible by pressing “c” or “m” keys, corresponding to the direction of the target arrow. If the target arrow points to the left, participants should press the “c” key; if it points to the right, they should press the “m” key. This task contains 100 trials including congruent (i.e., > > > > > or < < < < <) and incongruent (i.e., < < > < < or > > < > >) arrows. Each array will be displayed for 300 ms, followed by a fixed inter-stimulus interval of 1400 ms. This task will measure the number of correct items and the reaction time (RT).

**Fig. 4:**
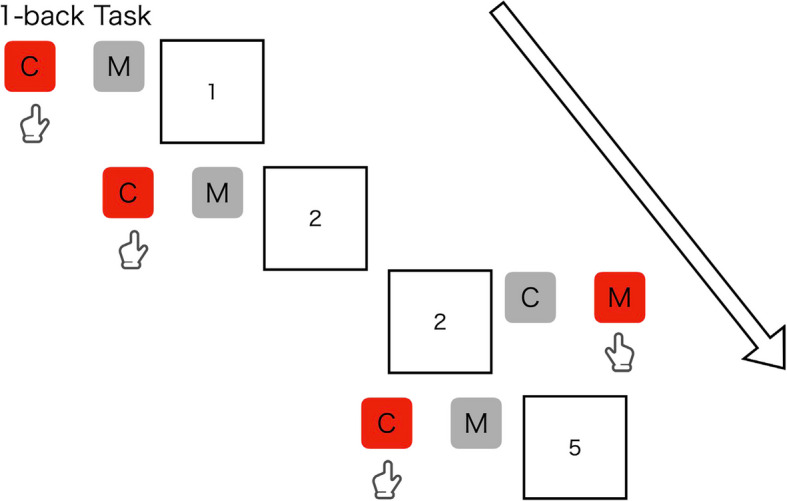
Examples of stimuli and correct judgments in 1-back task

**Fig. 5:**
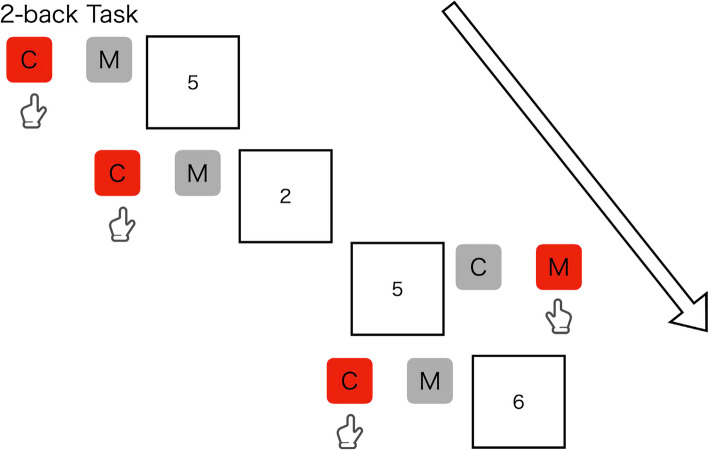
Examples of stimuli and correct judgments in 2-back task

The N-back task is commonly used to evaluate the working memory. This study employs computer-based 1-back (Fig. 4) and 2-back (Fig. 5) tasks, displaying numbers from one to nine on the display. Participants will need to respond with “c” and “m” keys according to a non-target and target and stimuli, respectively. In the 1-back task, participants will be instructed to respond with the “m” key as a target when a number appears as the same number presented one trial ago, and otherwise to respond with the “c” key as a non-target. In the 2-back task, participants will be instructed to respond with the “m” key as a target when a number appears as the same number presented two trials ago, and otherwise to respond with the “c” key as a non-target. The two tasks (i.e., one-back and two-back) consist of 90 trials with a 30% probability of the target appearing in each trial for all tasks, presenting a fixation for 2000 ms, followed by presenting a stimulus of number for 500 ms. Participants will be instructed to respond as quickly and accurately as possible. This task measures the number of correct items and RT.

**Fig. 6:**
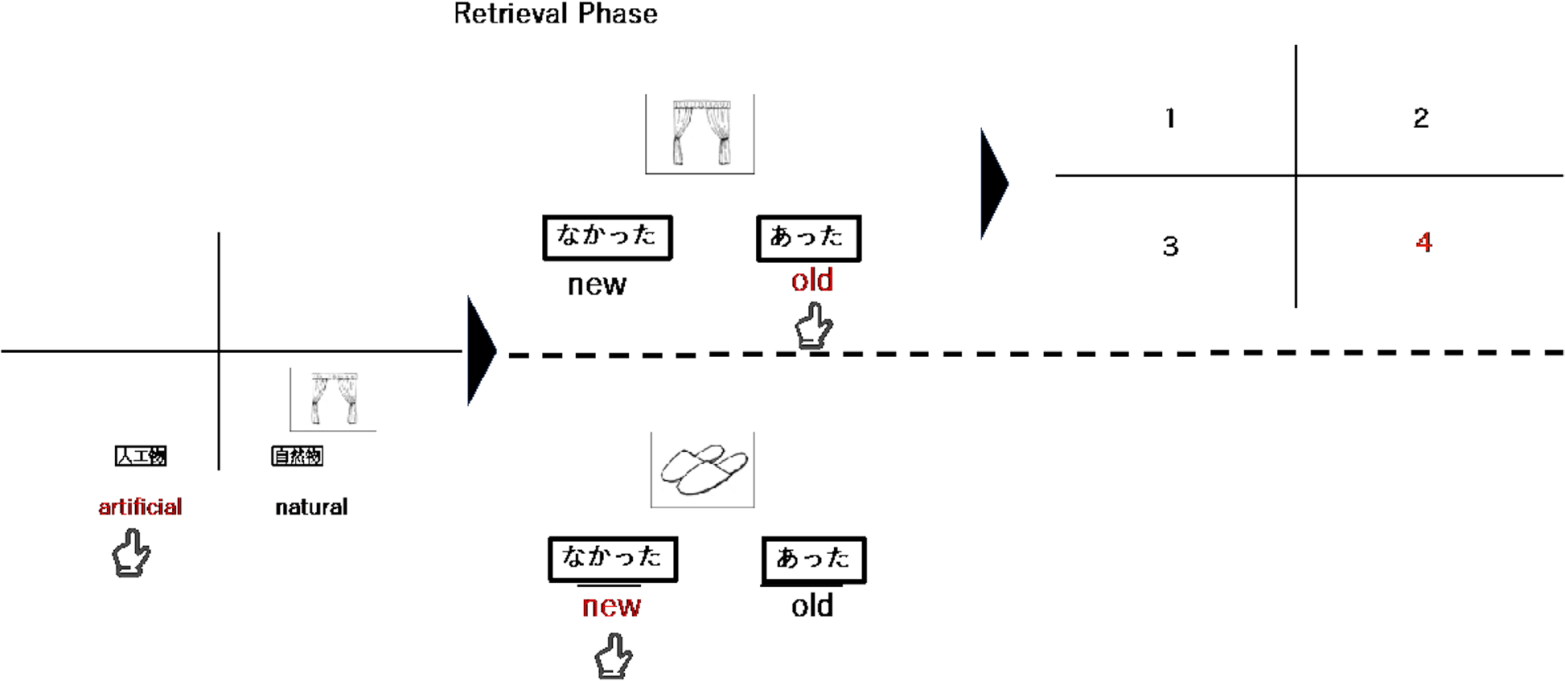
Examples of stimuli and correct judgments in the encoding and retrieval phase in Source memory task

The source memory task, which includes the encoding and retrieval phases, will be administered using a computer-based implementation (Fig. 6). The task paradigm is developed according to a procedure described in a previous study [49]. During the encoding phase, participants will be asked to memorize the presented white and black picture. They will also be asked to judge whether the picture depicts a natural (e.g., fruit and animal) or an artificial object (e.g., car and house). The pictures appear randomly on a display divided into four equal sections, both vertically and horizontally, with a grit layout line. The stimuli of the picture will be presented for 1500 ms, followed by an equal section interval of 2500 ms. The participants will be instructed to respond as accurately and quickly as possible. In the retrieval phase, the participants will be asked to press two keys (c: new, m: old) corresponding to whether the presented picture is old or new. When participants judge the picture to be old, the grid layout line divided into four equal sections will appear. Participants will need to press four keys (i.e., v, f, n, j) corresponding to where the old picture was presented. In total, 120 black-and-white outline picture are selected from a previous study [50]. For the encoding and retrieval phases, 108 pictures are selected, including three sets (i.e., 36 pictures per set) of equationally distributed natural and artificial objects. During the encoding phase, 72 images will be displayed. Thirty-six additional images will be presented during the retrieval phase. The remaining 12 images will be used in the practical phase of the task.

### Psychological characteristics

Grit will be measured using the Japanese translation of the 8-items short Grit Scale [51]. This scale was originally developed in English by Duckworth and Quinn [52]. Participants will be asked to respond to each item (e.g., “I often set a goal but later choose to pursue a different one”) on a 5-point Likert scale, with higher scores indicating superior grit ability.

Resilience will be measured using the 6-item Brief Resilience Scale-Japanese version [53]. The scale was originally developed and validated in English by Smith and Dalen [54]. Participants will be instructed to respond to each item (e.g., “I tend to bounce back quickly after hard times”) on a 5-point Likert scale. A higher score indicates superior resilience.

Mindset will be measured using the Japanese version of the Self-Theories of Intelligence Scale [55], which is a modified version based on the theory of self-regulation by Dweck and Yeager [56]. Within this framework, mindsets are categorized into two distinct types: fixed and growth mindsets. Individuals with a fixed mindset believe that intelligence is innate and immutable, whereas those with a growth mindset perceive intelligence as something that can be developed through continuous effort. Participants in this study will be asked to respond to scale items using a 5-point Likert scale, with higher scores indicating a stronger inclination toward a growth mindset. This scale measures the degree to which individuals agree with statements reflecting the flexibility and malleability of their intelligence.

### Physical activity

The Global Physical Activity Questionnaire (GPAQ) will be used to assess physical activity during a typical week [57]. The GPAQ categorizes physical activity into three domains: work-related, transport-related, and leisure or recreational. This questionnaire quantifies physical activity in terms of Metabolic Equivalent Tasks (METs). Moderate-intensity activities are equivalent to 4 METs, while vigorous-intensity activities correspond to 8 METs. Based on these assessments, the GPAQ classifies physical activity levels into three categories (low, moderate, and high) to provide a comprehensive overview of an individual’s physical activity profile.

### Mood

Mood will be assessed using the Profile of Mood States (POMS) 2nd edition [58]. The POMS enables the measurement of Tension-Anxiety, Depression-Dejection, Anger-Hostility, Vigor-Activity, Fatigue-Inertia, Confusion-Bewilderment, and Friendliness. Participants will be instructed to respond to each item on a 5-point Likert scale.

### Constipation

Constipation Assessment Scale will be used to assess changes in bowel habits [59]. The self-assessment questionnaire consists of eight items rated on a 3-point Likert scale. Higher scores indicate a tendency for constipation.

### Sleep

The sleep state will be assessed using the Japanese version of the Pittsburgh Sleep Quality Index (PSQI) [60]. This scale was originally developed by [61]. The self-report questionnaire comprises 10 question categories based on sleep quality, sleep latency, sleep duration, habitual sleep efficiency, sleep disturbances, use of sleeping medication, and daytime dysfunction. A higher PSQI score indicates severe sleep problems.

### Quality of life (QOL)

This study will use the MOS Short-Form 36-Item Health Survey to assess health status in terms of both physical and mental dimensions [62]. This will enable a comprehensive assessment of participants’ health status by assessing their physical function, role-physical, bodily pain, general health perception, validity, social functioning, role-emotional, and mental health. The physical component comprises physical functioning, physical role, bodily pain, and general health perceptions, whereas the mental component includes vitality, social functioning, emotional role, and mental health. The results of these assessments are indicative of the QOL and offer insights into how individuals perceive and experience their health status.

### Mental health

Perceived stress will be measured using the 10-item Japanese version of the Perceived Stress Scale [63]. The scale was developed and validated by Cohen, Kamarck [64]. Participants will need to respond to each item, scored from 0 to 4. Higher scores indicate a greater degree of perceived stress.

Depression levels will be assessed using the Japanese version of the 6-item Kessler Psychological Distress [65]. Participants will need to respond to each item, scored from 0 to 4 (e.g., 0 = all of the time and 4 = none of the time). Higher scores indicate a greater level of depression.

### Gut microbiota

Participants will be instructed to collect fecal samples at home using the Metabolokeeper® preservation solution (TechnoSuruga Laboratory, Shizuoka, Japan) before the pretest and after completing the intervention period. Each participant will be provided with a collection kit containing a sterile collection tube prefilled with 5 mL of Metabolokeeper. All fecal samples will be sent to Technosuruga Laboratory Co., Ltd. for analysis of gut microbiota composition. The sample using a microbiome analysis reveals α and β diversities, as well as levels of SCFAs. The analysis will involve extracting DNA from fecal samples using an amplicon sequencing method. The DNA will then be amplified by polymerase chain reaction (PCR). Subsequently, the V3-V4 region of the 16S ribosomal RNA (rRNA) region will be targeted for sequence analysis using the Illumina MiSeq system, and the data is processed using QIIME2 software [66]. The taxonomic assignment of representative sequences will be performed using the Greengenes database [66]. Additionally, a physicochemical analysis of the fecal samples will be performed to detect SCFAs based on the metabolism of dietary fibers and carbohydrates by the gut microbiota.

### Factors of aging control

The proteome analysis method to be employed in this study will allow for the quantitative measurement of proteins. This method utilizes a technology based on aptamers designed to exhibit specific affinity toward target molecular structures, thereby enabling the detection of various protein configurations. Proteomic analysis can measure approximately 1000 different types of proteins. The analysis in this study will primarily reveal the cytokines related to the immune system. Furthermore, we will focus on identifying key proteins relevant to our research, including mTOR and SIRT, which are recognized as factors involved in the control of aging. For this purpose, blood samples from the participants will be analyzed using proteomic analysis.

### Physical function

To assess physical function, this study will measure hand grip strength, the 5-m walk test, and single-leg support tests. Participants will be asked to stand up and sit down from a chair five times as quickly as possible. Hand grip strength is considered an indicator of health status [67, 68]. Muscle strength is a diagnostic criterion for sarcopenia, characterized by a decline in muscle mass and strength with age. Previous studies have shown that hand grip strength is correlated with cognitive function in older adults [69]. It has been suggested that a decline in hand grip strength may lead to cognitive impairments during aging. The time taken to complete the five repetitions will be recorded. The participants will also be instructed to walk a distance of 5 m at their maximum walking speed. The time required to cover the distance will be recorded. Single-leg support tests will be performed twice for each leg, with the eyes open and closed. The duration for which the participant can maintain the stance will be recorded, with a maximum duration of 60 s.

### Plans to promote participant retention and complete follow-up {18a}

The training menu records will be provided as feedback to each participant in the intervention group to encourage participation by highlighting their cumulative efforts. In cases where participants are unable to attend due to urgent matters or illness, the responsible researchers will coordinate alternative attendance dates via email or telephone. Furthermore, to enhance participant retention, reminder emails will be sent regularly to encourage participants to participate in the upcoming week’s class.

### Data management {19}

The collected data will be anonymized, and signed consent forms will be stored in a locked cabinet for 5 years following the completion of the study. Electronic data will be saved on a secure site, accessible only within the campus network, and stored on a high-capacity, password-protected hard drive. In the event of a participant dropping out, we will specify the reason and report it.

### Confidentiality {27}

Each participant will be assigned a unique ID. The cross-reference table, linking names to ID codes, will be stored securely on campus without permission to be removed. Electronic data, including personal information, will be locked using passwords. This personal information, capable of identifying individuals, will not be included in the database for statistical analysis or disclosed in any conference presentations or publications.

### Access to data {29}

Owing to a conflict of interest, one author (KS) is prohibited from accessing the data analysis and management. MT is responsible for the statistical analysis. All other authors, except KS, will be able to access the dataset.

### Plans for collection, laboratory evaluation, and storage of biological specimens for genetic or molecular analysis in this trial/future use {33}

Participants will undergo blood sampling before and after the study. Approximately 10 ml of blood sample will be obtained. The collected samples will be analyzed for complete blood count, including red blood cell (RBC) count, hemoglobin (Hb), hematocrit (Hct), mean corpuscular volume (MCV), mean corpuscular hemoglobin (MCH), mean corpuscular hemoglobin concentration (MCHC), platelet count, and white blood cell count (WBC), as well as blood metabolites. These blood samples will also be used for omics analysis.

The blood used for proteome analysis will be processed using a centrifuge to separate serum and plasma. The serum will be discarded, and the plasma will be stored at − 80 °C in a secure, temperature-controlled environment at Tohoku University for a period of 5 years following the completion of the study. After 5 years, all stored plasma specimens will be systematically destroyed in accordance with institutional guidelines and relevant regulations to ensure the protection of participant confidentiality and data integrity. Fecal samples will be sent to TechnoSuruga Laboratory Co., Ltd. for comprehensive gut microbiome analysis. After the completion of the analysis, all fecal specimens will be disposed of properly.

### Statistical methods

#### Statistical methods for primary and secondary outcomes {20a}

The statistical analyses will be performed using Python, R, Stata, and Mplus. The significance threshold for all statistical analyses will be set at *p* < 0.05. When the statistical analysis does not find statistical significance for our outcome based on our hypothesis, a 95% confidence interval will be used to estimate the effects of exercise. For cognitive performance, aging control factors, specific gut bacteria, and linear mixed model (LMM) analyses will be performed, including age and sex as covariates. The primary outcomes will be assessed as follows:

### Primary outcomes

1. Cognitive function
  ◦ We will use the same standardized cognitive assessments outlined previously.
  ◦ Analysis: LMM comparing changes in cognitive scores from baseline to 16 weeks after the intervention between the exercise and control groups, adjusting demographics (age and sex).
2. Gut microbiota/metabolite
  ◦ Measures: α- and β-diversity, relative abundance of key bacterial taxa, and SCFA concentrations.
  ◦ Analysis: LMM for α and β-diversity/ metabolite (i.e., SCFA) changes from baseline to 16 weeks after the intervention. Baseline values will be included as covariates.
3. Aging-related proteins
  ◦ Targets: SIRT, mTOR, and other proteins relevant to aging control (aptamer-based detection).
  ◦ Analysis: LMM to assess changes in protein expression levels from baseline to 16 weeks after the intervention, comparing intervention vs. control, with baseline adjustment.

As for the exploratory approach regarding gut microbiota and proteins in omics analysis, linear model analysis with the Elastic Net approach (e.g., the Lasso and Ridge approaches) will be used. This approach helps select the most appropriate regression model for the data. As for psychological characteristics and mental health, structural equation modeling (SEM) will be performed. Furthermore, in each group, a multigroup analysis will be conducted to reveal the effects of circuit training exercises on these psychological factors. The secondary (exploratory) outcomes will be assessed as follows:

1. Comprehensive proteomics and cytokine analysis
  ◦ We will apply an Elastic Net approach to ~ 1000 proteins to identify which markers show the largest change in response to the exercise intervention.
  ◦ Markers identified by Elastic Net as differentially expressed will be further analyzed to determine their relationship with changes in cognitive function and gut microbiota measures.
2. Mediation/path analysis (SEM)
  ◦ We will use structural equation modeling (SEM) or mediation models to test whether changes in specific proteins or gut microbiota mediate the relationship between the exercise intervention and improvements in cognitive function.
  ◦ For instance, if SCFA-producing bacteria increase in the exercise group, we will examine whether these changes lead to alterations in SIRT/mTOR signaling, which subsequently improve cognitive performance.
3. Psychological and physical assessments
  ◦ Grit, Resilience, Mindset, Depression, Stress, Quality of Life, and physical function measures will be incorporated into SEM analysis to examine how these psychological factors may mediate exercise-induced cognitive improvements.

In our primary analysis, an LMM is conducted to examine cognitive performance, gut microbiota, and aging control factors. As a secondary analysis, we will perform a linear model analysis using the Elastic Net approach for omics analysis and SEM to further explore the relationships among exercise, protein expression, psychological characteristics, mental health, and Quality of Life (QOL). Specifically, we will employ SEM to examine both the direct effect of exercise on cognitive function and explore potential indirect (mediating) effects through grit and resilience measures. We will randomly assign participants into exercise vs. control groups in a 1:1 ratio, ensuring that baseline variability in gut microbiota, cytokines, and protein levels is minimized across groups. Specifically,

- Randomization will be used to minimize systematic bias in baseline cognitive function, microbial and proteomic profiles.
- Baseline adjustments:
  - ◦ We will apply a linear mixed model with random intercept for adjustment of baseline conditions. Since this study will employ a randomized study design, baseline differences may be attributed to chance. For example, randomized intervention studies used a linear mixed model for assessing the effects of exercise on cognitive function [70–72] without adjustment for baseline conditions

By combining randomization with rigorous statistical adjustment, we can isolate the effects of the exercise intervention on outcome changes and reduce confounding from individual differences in biomarker levels.

Although the trial is open-label, the data analyses will be conducted in a partially blinded manner. While participants and investigators will not be blinded due to the nature of the interventions, the statisticians will receive de-identified datasets with coded treatment assignments (e.g., “Treatment A” and “Treatment B”) to minimize potential bias. The treatment codes will only be revealed after the completion of the primary analyses.

### Interim analyses {21b}

The investigation of this study will be completed in one phase.

### Methods for additional analyses (e.g., subgroup analyses) {20b}

Subgroup analyses will be conducted based on age and sex. Furthermore, as we collect additional data on physical activity through a global physical activity questionnaire (GPAQ), a subgroup analysis will be conducted on the relationship between daily life physical activities and the study outcomes.

### Methods in analysis to handle protocol non-adherence and any statistical methods to handle missing data {20c}

In this study, we will conduct an intention-to-treat analysis, including data from all participants, even those who drop out. To address missing values, we will apply the multiple imputation method and a machine learning approach, such as random forest.

### Plans to give access to the full protocol, participant-leveldata, and statistical code {31c}

All data and statistical codes can be provided by the corresponding author upon request. In this case, personal details will be anonymized and cannot be tracked from the provided dataset.

### Oversight and monitoring

#### Composition of the coordinating center and trial steering committee {5d}

The study protocol and researchers involved are under the supervision of the Ethics Committee of Tohoku University Graduate School of Medicine. The principal investigator will manage the study implementation and collected datasets. The principal investigator’s responsibilities include participant recruitment, ensuring data quality, and participant follow-up. Any modifications to the study protocol or occurrence of adverse events will be promptly reported to the Ethics Committee. The study team convenes weekly meetings to discuss the trial’s progress.

### Composition of the data monitoring committee, its role, and reporting structure {21a}

There are no specific committees for data monitoring. The researchers responsible report any issues and adverse events to the Ethics Committee through monitoring processes.

### Adverse event reporting and harms {22}

Adverse events must be reported to an Ethics Committee. Representative researchers are required to confirm the details of each event. These events are graded according to the Japanese version of the National Cancer Institute Common Terminology Criteria (CTCAE) for Adverse Events v4.0. If an adverse event occurs, the researcher must immediately contact the medical staff to ensure prompt and appropriate treatment of the affected participant. Based on the study population and intervention characteristics, anticipated harms include fatigue, dizziness, and headache, which will be systematically monitored through participant self-reports. Any unexpected adverse events will be recorded on an as-occurs basis and reported as necessary, with collection occurring non-systematically through spontaneous participant reporting or clinician detection. For reporting purposes, all adverse events of CTCAE grade 3 or higher will be documented in full, while lower-grade adverse events will be reported based on their frequency and clinical relevance.

### Frequency and plans for auditing trial conduct {23}

The Ethics Committee is supervising this trial, as previously mentioned. However, plans for audit are not integrated into this study. When the study protocol changes or adverse events occur, the researchers will report the results to the Ethics Committee.

### Research ethics approval {24}

This study was approved by the Ethics Committee of Tohoku University School of Medicine (research ID: 2023–1–1016). Our study design was registered with the University Hospital Medical Information Network (UMIN-CTR, UMIN000053937).

### Plans for communicating important protocol amendments to relevant parties (e.g., trial participants, ethical committees) {25}

Any modifications to the study protocol are reported to the Ethics Committee and require their approval. Documents relevant to informed consent will reflect the modifications. Furthermore, if necessary, the principal investigator will notify the participants about the modifications.

### Dissemination plans {31a}

The findings of this study will be presented in peer-reviewed journals and at academic conferences.

## Discussion

This study provides the first insights into the effects of long-term circuit training on cognitive function through comprehensive perspectives on the gut microbiota, cytokines, and proteins involved in aging control. As a secondary aim, we identify the factors associated with the beneficial effects of exercise on cognitive function. Furthermore, this study examines the relationship between the beneficial effects of exercise, psychological characteristics, and mental health. Although most studies have highlighted the benefits of long-term exercise on cognitive function, they have not explored these broader perspectives. The potential impact of these benefits encourages engagement in physical activity for brain and mental health, particularly in the aging population. Clarifying these effects and relationships could pave the way for further understanding of the potential effects of exercise on cognition.

Considering the relationship between exercise and gut microbiota, long-term exercise modulates the environment of the gut microbiota and creates appropriate environments to promote the secretion of growth factors, such as BDNF [73]. This study aims to unveil the association between exercise-induced modulation of the gut microbiota and growth factors. Growth factors have also been suggested to stimulate aging control [7]. The activation of mTOR and SIRT has been proposed to promote neural plasticity [7, 74], potentially leading to improvements in cognitive performance. Thus, the beneficial effects of circuit training on cognitive function may be rooted in the communication between the gut, aging control factors, and growth factors.

From a different perspective, Nouchi and Nouchi [75] discovered that circuit training exercises increase vigor-activity mood scores in middle-aged and older populations. Thus, based on a previously established association, long-term combination exercise may alter psychological characteristics and improve mental health [76–78]. Furthermore, it is expected that the gut microbiota and factors controlling aging are involved in psychological characteristics and mental health. This study plans to use SEM analysis to unravel the complex relationships between exercise, gut microbiota, aging control factors, cytokines, and proteins involved in neuroplasticity and aging control, psychological characteristics, mental health, and QOL. Elucidating these causal relationships can help reveal the potential effects of circuit training exercises on aging populations.

Nonetheless, a notable limitation of this study is the lack of dietary intake monitoring. While this approach reflects a real-world setting where participants maintain their usual dietary habits during the exercise intervention, it may limit our understanding of the relationship between exercise and changes in gut microbiota. Future research should consider incorporating dietary controls to better understand how dietary patterns might influence the gut microbiota response to exercise.

Our findings advance the current understanding of exercise-induced cognitive enhancement in several important ways. First, we integrate exercise science, gut microbiota analysis, and aging research to demonstrate how shorter-duration circuit training (30 min) can effectively promote cognitive health through multiple physiological pathways. Second, our exploratory analyses of gut microbiota and protein expression provide novel insights into the potential mechanisms through which circuit training may influence cognitive function, opening new avenues for future research in exercise neuroscience. Finally, by delineating the relationship between exercise adherence, grit, and resilience using SEM, we offer practical implications for developing sustainable exercise interventions that can be readily adopted in daily life. These insights could inform future intervention studies aimed at preventing cognitive decline in aging populations while ensuring practical feasibility in real-world settings.

In summary, this study aims to clarify the effects of long-term circuit training exercises on executive and memory functions from a comprehensive perspective. These findings address age-related cognitive decline from the perspective of exercise. In developed countries, including Japan, cognitive decline exerts upward pressure on healthcare costs. Additionally, diminished cognitive function is likely linked to deterioration of mental health and QOL. Although numerous studies have suggested that long-term exercise is a preventive tool against cognitive decline, the comprehensive relationship between exercise, the gut, aging, and the brain remains unclear. To date, the beneficial effects of exercise have not been sufficiently emphasized to encourage the middle-aged and older populations to engage in exercise to improve their brain health. Initiating physical exercise is a significant obstacle, particularly in sedentary populations. We have designed our study protocol considering these factors. We believe that our findings will contribute to addressing the issue of cognitive decline in aging populations by providing valuable insights and potentially guiding interventions.

## Trial status

Patient recruitment began in June 2024 and is anticipated to be completed by February 2025. The current protocol is version 2.0. This trial is currently underway in our laboratory.

## Supplementary Information

Supplementary Material 1.

## Data Availability

The authors, except for KS, will have access to the dataset generated during the trials. The dataset will be available upon logical requests according to ethical approval.

## Abbreviations

BDNF: Brain-derived neurotrophic factor
IGF-1: Insulin-like growth factor
mTOR: Mechanistic target of rapamycin
SIRT: Sirtuin genes
SCFAs: Short-chain fatty acids
HR: Heart rate
SEM: Structural equation modeling
LMM: Linear mixed model
GPAQ: Global Physical Activity Questionnaire
POMS: Profile of Mood States
QOL: Quality of life
