# Supplementary material for: Circuit training intervention for cognitive function, gut microbiota, and aging control: study protocol for a longitudinal, open-label randomized controlled trial
Source: Trials. 2025 Mar 18;26:94. doi: 10.1186/s13063-025-08807-9 (PMC11917102; doi:10.1186/s13063-025-08807-9)
Supplement: Supplementary file 1 — Supplementary Material 1. [file 13063_2025_8807_MOESM1_ESM.docx]

Supplemental Table 1. Additional WHO Trial Registration Data Set Items

| Data Category | Item Details |
| --- | --- |
| Summary Results | Not yet available. Results will be reported after the study is completed in accordance with the CONSORT guidelines and published in a peer-reviewed journal. |
| IPD Sharing Statement | We do not plan to share individual participant data from this study. |

Note: Trial Registration: UMIN-CTR (UMIN000053937; registered on March 21, 2024)
